# Enhanced amphiregulin exposure promotes modulation of the high grade serous ovarian cancer tumor immune microenvironment

**DOI:** 10.3389/fphar.2024.1375421

**Published:** 2024-05-20

**Authors:** Jasmine Ebott, Julia McAdams, Chloe Kim, Corrine Jansen, Morgan Woodman, Payton De La Cruz, Christoph Schrol, Jennifer Ribeiro, Nicole James

**Affiliations:** ^1^ Women and Infants Hospital, Department of Obstetrics and Gynecology, Program in Women’s Oncology, Providence, RI, United States; ^2^ Department of Obstetrics and Gynecology, Warren-Alpert Medical School of Brown University, Providence, RI, United States; ^3^ School of Public Health, Brown University, Providence, RI, United States; ^4^ Department of Molecular Biology, Cell Biology, and Biochemistry, Brown University, Providence, RI, United States; ^5^ Pathobiology Graduate Program, Brown University, Providence, RI, United States

**Keywords:** amphiregulin (AREG), high-grade serous ovarian cancer, tumor immune microenvioronment, immunosupperssion, chemoresistance

## Abstract

High grade serous ovarian cancer (HGSOC) is a lethal gynecologic malignancy in which chemoresistant recurrence rates remain high. Furthermore, HGSOC patients have demonstrated overall low response rates to clinically available immunotherapies. Amphiregulin (AREG), a low affinity epidermal growth factor receptor ligand is known to be significantly upregulated in HGSOC patient tumors following neoadjuvant chemotherapy exposure. While much is known about AREG’s role in oncogenesis and classical immunity, it is function in tumor immunology has been comparatively understudied. Therefore, the objective of this present study was to elucidate how increased AREG exposure impacts the ovarian tumor immune microenvironment (OTIME). Using NanoString IO 360 and protein analysis, it was revealed that treatment with recombinant AREG led to prominent upregulation of genes associated with ovarian pathogenesis and immune evasion (*CXCL8*, *CXCL1*, *CXCL2*) along with increased STAT3 activation in HGSOC cells. *In vitro* co-culture assays consisting of HGSOC cells and peripheral blood mononuclear cells (PBMCs) stimulated with recombinant AREG (rAREG) led to significantly enhanced tumor cell viability. Moreover, PBMCs stimulated with rAREG exhibited significantly lower levels of *IFNy and IL-2*. *In vivo* rAREG treatment promoted significant reductions in circulating levels of IL-2 and IL-5. Intratumoral analysis of rAREG treated mice revealed a significant reduction in CD8^+^ T cells coupled with an upregulation of PD-L1. Finally, combinatorial treatment with an AREG neutralizing antibody and carboplatin led to a synergistic reduction of cell viability in HGSOC cell lines OVCAR8 and PEA2. Overall, this study demonstrates AREG’s ability to modulate cytotoxic responses within the OTIME and highlights its role as a novel HGSOC immune target.

## Introduction

High grade serous ovarian cancer (HGSOC) is the most lethal of all gynecologic malignancies with a 5-year survival rate just below 50%, due to the fact that patients are frequently diagnosed at an advanced stage and possess high recurrence rates 12–18 months after initially achieving remission (Luvero et al., 2019; Macchia et al., 2023). Furthermore, recurrent HGSOC tumors are heavily chemoresistant and therefore do not always respond to traditional platinum-taxane based chemotherapies that are utilized in the frontline setting. In recent years, targeted approaches such as the anti-angiogenic therapy bevacizumab and the poly (ADP-ribose) polymerase (PARP) inhibitor olaparib have been implemented in the maintenance setting as standard of care for HGSOC patients. However, with the exception of *BRCA1/2* mutated patients who significantly benefit from olaparib treatment, these targeted therapies have not had profound effects on overall HGSOC survival rates (OMalley et al., 2023). In addition, the majority of HGSOC patients derive no significant benefit from clinically available immunotherapies, as numerous clinical trials have demonstrated low response rates to programmed cell death protein 1 (PD-1) based therapies (James et al., 2020), despite the fact that intratumoral T cells are known to be highly prognostic in ovarian cancer (Zhang et al., 2003; Hwang et al., 2012). Hence, it has been theorized that the muted response to clinically available immunotherapies can be attributed to the uniquely immunosuppressive ovarian tumor immune microenvironment (OTIME), which is composed of high levels of T regulatory cells (Tregs), adipose tissue, and cancer associated fibroblasts (CAFs) that collectively contribute to tumor immune evasion and further drive ovarian pathogenesis (James et al., 2020).

In an effort to identify novel immune targets that are more representative of the unique OTIME, we previously performed a genomic analysis in matched diagnostic biopsy and interval debulking HGSOC patient tissue, obtained both prior to and following neoadjuvant chemotherapy (NACT) exposure to characterize OTIME adaptations (James et al., 2022). This analysis revealed that the gene amphiregulin (AREG) exhibited the highest fold-upregulation of out a panel of 770 of the most commonly studied immune oncology genes. AREG is a secreted glycoprotein and low-affinity epidermal growth factor receptor (EGFR) ligand and has an established role in promoting ovarian cell proliferation, metastasis, cancer stemness, and therapy resistance in ovarian cancer (Cheng et al., 2016; Tung et al., 2017). Furthermore, in classical immunity, AREG is thought to function as a Th2 cytokine that controls inflammation and downregulates adaptive immune responses (Zaiss et al., 2013; Zaiss et al., 2015; Singh et al., 2022). However, there are limited studies evaluating AREG’s role in tumor immunology. Therefore, in this current investigation we sought to begin to elucidate the impact of AREG on multiple aspects of the OTIME.

## Methods

### Cell culture

HGSOC cell lines PEA1/PEA2 cells were obtained from Millipore Sigma and cultured in RPMI 1640 supplemented with 2 mM Glutamine, 2 mM Sodium Pyruvate, and 10% Fetal Bovine Serum (FBS) and 1% penicillin/streptomycin. OVCAR8 HGSOCs were obtained from American Type Culture Collection (ATCC) and ID8 p53^−/−^ cells were generously gifted by the Freiman lab at Brown University that were originally generated by the McNeish lab at the University of Glasgow. Both OVCAR8 and ID8 p53^−/−^ cells were cultured in Dulbecco Modified Eagle Medium (DMEM) supplemented with 10% FBS and 1% penicillin/streptomycin. All cells were kept in a 37°C/5% CO_2_ humidified chamber. Cells were treated with 200 ng/mL human recombinant AREG (rAREG; R&D Systems, 262-AR-100) or with BSA control at various timepoints (15 min- 4 h). HGSOC cells were treated with 10 μM of ruxolitinib (Selleckchem, S1378) or DMSO control (Sigma Aldrich, D54879) for 48 h.

### RNA isolation and NanoString nCounter^®^ PanCancer IO360

OVCAR8 and PEA1 cells were stimulated with 200 ng/mL of rAREG or BSA control for 2 h. RNA isolation was performed using the Trizol extraction/LiCl high salt precipitation and NanoString nCounter^®^ PanCancer IO360 was performed as previously described in detail (James et al., 2022). A total of three biological replicates per treatment in each cell line were submitted for analysis.

### NanoString nCounter^®^ PanCancer IO360 analysis

Data was analyzed in nSolver Advanced Analysis software and ROSALIND^®^ (https://rosalind.bio/), with a HyperScale architecture developed by ROSALIND, Inc. (San Diego, CA). The QC step generated read distribution percentages, violin plots identify heatmaps, and sample MDS plots. Normalization, fold changes and *p*-values were calculated using criteria provided by NanoString^®^ (https://nanostring.com). Control and rAREG samples were used to construct groups, respective to each cell line. ROSALIND^®^ follows the nCounter^®^ Advanced Analysis protocol of dividing counts within a lane by the geometric mean of the normalizer probes from the same lane. Housekeeping probes to be used for normalization are selected based on the geNorm algorithm as implemented in the NormqPCR R library (Perkins et al., 2012)^.^ Fold changes and *p*-values are calculated using the fast method as described in the nCounter^®^ Advanced Analysis 2.0 User Manual Document Library (nanostring.com). The Benjamini-Hochberg method of estimating false discovery rates (FDR) was used to adjust *p*-values. The clustering of genes for the final heatmap of differentially expressed genes was performed using the Partitioning Around Medoids (PAM) method using the fpc R library (Hening, 2024) that takes into the account the direction and type of all signals on the pathway, the position, role and type of every gene, etc. Hypergeometric distribution was employed to analyze the enrichment of pathways, gene ontology domain structure, and other ontologies. The topGO R library (Alexa and Rahnenfuher, 2019) was employed to determine local similarities and dependencies between GO terms in order to perform Elim pruning correction. Interpro (Mitchell et al., 2019), NCB (Geer et al., 2010), MSigDB (Subramanian et al., 2005; Liberzon et al., 2011), REACTOME (Fabregat et al., 2018), and WikiPathways (Slenter et al., 2018) databases were referenced for enrichment analysis. Enrichment was calculated relative to a set of background genes relevant to this experiment. RCC files were deposited in NCBI’s Gene Expression Omnibus (GEO) (Edgar et al., 2002) and are accessible through GEO series accession number GSE252495.

### RNA isolation and quantitative PCR

RNA isolation and quantitative PCR was performed as previously described (James et al., 2022). Validated human primers were purchased from Bio-Rad (*CXCL1*, *DUSP5*, *IL-11*, *CXCL2*, *IL6*, *IFNy*, *IL-2*, *GZMB*). Custom primer sequences (Invitrogen) are as follows:

18s rRNA-F-CCGCGGTTCTATTTTGTTGG

18s rRNA-R-GGCGCTCCCTCTTAATCATG

### Phosphoproteomics

OVCAR8 and PEA1 cells were treated with 200 ng/mL of rAREG or BSA control for 15 min, and then protein was collected in lysis buffer supplied by the Proteome Profiler Human Phospho-Kinase Array Kit (R&D Systems, ARY003C). Manufacturer’s instructions were followed and membranes were developed using the Bio-Rad ChemiDoc Imaging System. ImageJ was employed to perform background subtraction and measure spot density.

### Western blot

Protein was extracted from cell pellets using Cell Lysis Buffer (Cell Signaling 9803) with 1 mM of a protease inhibitor cocktail (AbCam, ab65621). Concentrations for all extracted proteins were determined by the DC Protein Assay (Bio-Rad Laboratories, 5000116). Equal amounts of proteins were boiled at 70°C with Novex Sample Reducing Agent (Life Technologies, NP009) and NuPAGE LDS sample buffer (ThermoFisher Scientific, NP0007) into a 4%–12% gradient SurPAGE™ Bis-Tris Gel (GeneScript, M00652). The gel was transferred using a semi-dry method to methanol activated PVDF membrane using the Trans-Blot Turbo RTA Transfer Kit PVDF (Bio-Rad, 1704273), Trans-Blot Turbo 5x Transfer Buffer (Bio-Rad, 10026938), and the Bio-Rad Trans-Blot Turbo Transferring System (1.3A-25V) for 10 min. Membranes were then blocked in 5% milk in phosphate-buffered saline with 0.05% Tween 20 (PBS-T) for 30 min at room temperature, and primary antibodies were incubated overnight at 4°C diluted in 5% milk in PBS-T. Secondary antibodies were then diluted in 5% milk in PBS-T for 1 h at room temperature. Membranes were washed with PBS-T in between primary and secondary incubations and following the secondary incubation. Clarity™ Western ECL substrate (Biorad, 102030779 [peroxide solution], 102030787 [luminol/enhancer solution]) was used to detect HRP-tagged secondary antibodies. The Bio-Rad ChemiDoc Imaging System was used to image all blots and GAPDH was employed as a loading control. All uncropped blots can be seen in Supplementary Material S1. Antibodies and dilutions were as follows:

STAT3 (Cell Signaling, 4904S, 1:500) or (Proteintech, 60199-1-1g, 1:500)

Phospho-STAT3 (Cell Signaling, 9145S, 1:500)

PD-L1(Proteintech, 66248-1-1g, 1:500)

GAPDH (Santa Cruz Biotechnology, 47724, 1:1,000)

ERK (Cell Signaling, 9102S, 1:500) or (Proteintech, 11257-1-AP, 1:500)

Phospho-ERK (Cell Signaling, 4376SS, 1:500)

AKT (Proteintech, 60203-2-1g, 1:500)

Phospho-AKT (Proteintech, 28731-1-AP, 1:500)

AREG (Protientech, 16036-1-AP, 1:500)

Anti-Rabbit (Cell Signaling, 7074S, 1:1,000)

Anti-Mouse (Cell Signaling, 7076S, 1:1,000)

### Enzyme-linked immunosorbent assay (ELISA)

OVCAR8 and PEA1 cells were treated with 200 ng/mL of rAREG or BSA control for 2 and 4 h. Their respective media was collected and secreted levels of IL-6 were examined using a commercially available IL-6 ELISA kit (ab178013). Media was diluted 4-fold using the kit provided Sample Diluent NS solution. Manufacturer’s instructions were followed with the endpoint reading at 450 nm. All samples were run in duplicate, with three biological replicates of each sample.

### Cell viability assays

#### HGSOC and peripheral blood mononuclear cell (PBMC) co-cultures

HGSOC cells were seeded in a 96-well plate (20,000 cells/well) and allowed to grow for 24-h. PBMCs (HumanCells Biosciences, PBMC-C10M) were co-cultured with HGSOC cells in a 5:1 ratio (James et al., 2019) and stimulated with 200 ng/mL of rAREG or BSA control. After 24 h, 10 μL/well of CellTiter 96^®^ Aqueous One Solution Cell proliferation MTS Assay (Promega, G3580), incubated for 1 h at 37°C/5% CO_2_, and finally read at 492 nm to assess cell viability.

#### AREG neutralizing antibody and chemotherapy treatments

PEA2 and OVCAR8 cells were seeded in a 96-well plate (20,000 cells/well) and allowed to grow for 24-h. Cells were pre-treated with carboplatin (400 μM for PEA2, 300 μM for OVCAR8; Santa Cruz Biotechnology, CAS 4157.5-94-4) or DMSO control (Sigma Aldrich, D54879) for 24-h, and with 30 μM of AREG neutralizing antibody (R&D Systems, MAB262-100) or corresponding IgG control (MAB002) for 48-h prior to cell viability assessment as described above.

#### Animals

C57BL/6 mice were purchased from Jackson Laboratories (strain#000664) All animal protocols were approved by the Brown University Animal Care and Use Committee (#22-09-0002) and performed in accordance with the National Institutes of Health Guide for the Care and Use of Laboratory Animals. This protocol was reviewed and acknowledged by the Lifespan University Institutional Animal Care and Use Committee (#505422).

#### *In vivo* treatment and tissue collection

7-week-old C57BL/6 mice were inoculated with five million ID8p53^−/−^ cells intraperitoneally (IP). 28-day post tumor inoculation, mice were treated with either rAREG (400 μg/kg; R&D Systems, 989-AR-CF) or saline, daily for a maximum of 6 days until large ascites formation, at which point mice were euthanized by carbon dioxide inhalation. Tissue was harvested and immediately fixed in a 1:10 formalin solution overnight and then placed in 30%, 50%, and 70% ethanol for 30 min each. Previously fixed tumors were then submitted to the Brown University Molecular Pathology Core for standard paraffin embedding and 5 μM serial sectioning.

#### Mouse ascites and serum multiplex assays

Ascites was collected from mice post-mortem and then spun at 5,000 g for 10 min at 4°C. Whole blood was collected via cardiac puncture post-mortem into serum separator tubes, allowed to clot for 30 min and then spun at 3,000 g for 15 min at 4°C. Both ascites supernatant and serum was collected and stored at −80°C. Ascites and serum from mice treated with rAREG (*n* = 5) and saline (*n* = 5) were analyzed using a Mouse Cytokine/Chemokine 44-Plex Discovery Assay^®^ Array (MD44) by Eve Technologies (Calgary, Canada), to simultaneously determine the levels of the of the following immune factors: Eotaxin, Erythropoietin, 6Ckine, Fractalkine, G-CSF, GM-CSF, IFNB1, IFNy, IL-1α, IL-1β, IL-2, IL-3, IL-4, IL-5, IL-6, IL-7, IL-9, IL-10, IL-11, IL-12p40, IL-12p70, IL-13, IL-15, IL-16, IL-17, IL-20, IP-10, KC, LIF, LIX, MCP-1, M-CSF, MDC, MIG, MIP-1α, MIP-1β, MIP-3α, MIP-3B, RANTES, TARC, TNFα, VEGF-A. Each analyte was bound to a differently colored/fluorescent bead to allow for simultaneous detection of all of the aforementioned immune factors in a single assay. A bead analyzer (Bio-Plex 200) first activates the fluorescent dye via laser, then excites the streptavidin-phycoerythrin fluorescent conjugate with a second laser, allowing for measurement of each specific analyte. Each sample was performed in duplicate.

#### Fluorescent immunohistochemistry

FFPE mouse tumors were baked for 2 h at 65°C and then washed in SafeClear xylene substitute, 100% ethanol, 95% ethanol, 70% ethanol, deoxygenated water, and FTA Hemagglutination buffer for 10 min at each wash on a shaker. Antigen retrieval was then performed via Antigen retrieval solution (1X; Vector Laboratories, H-3300) and heated at 95°C for 20 min. Slides were blocked in 5% horse serum diluted in FTA Hemagglutination buffer and incubated overnight in primary antibody at 4°C. Secondary antibody was then added for 1 h in the dark at room temperature. Between each step slides were washed with FTA Hemagglutination buffer. Lastly, slides were cover-slipped with DAPI containing mounting medium (Vector Laboratories, H-1200). Primary and secondary antibodies and respective dilutions were as follows:

CD8 (Proteintech, 29896-1-AP, 1:50)

PD-L1 (Proteintech, 66248-1-1g, 1:50)

CD4 (Proteintech, 677886-1-1g, 1:50)

CD45 (Proteintech, 20103-1-AP, 1:50)

CD45 (Proteintech, 67786-1-1g, 1:50)

Anti-Rabbit DyLight™488 (Vector Laboratories, DI-1488, 1:1,000)

Anti-Mouse DyLight™594 (Vector Laboratories, DI-2594, 1:1,000)

#### Image analysis

For PD-L1 intensity and CD8+/CD4+ T cell counts, three and five randomly selected fields per case were selected based on DAPI staining, respectively. Images were acquired via a spinning disk confocal Nikon Eclipse Ti microscope at a ×20 objective. Image processing and analysis was performed utilizing ImageJ. For PD-L1 staining analysis, images were thresholded for specific staining and mean intensity was calculated. For CD8^+^ and CD4^+^ T cells, the total number of positive cells co-stained with CD45 and DAPI were counted. Representative images were taken at ×20 or ×40.

#### Statistical analysis

Statistical analyses were performed in GraphPad Prism. Student t-tests were performed to determine differences in control and rAREG treated cell lines and mice. All *p*-values reported with the exception of ROSALIND NanoString Analysis were 2-tailed and unadjusted.

##### cBioPortal

cBioPortal (Cerami et al., 2012; Gao et al., 2013) was used to analyze TCGA ovarian serous cystadenocarcinoma cohorts from the Firehose Legacy (*n* = 617) or Nature 2011 (*n* = 489) studies. AREG’s association with platinum status (Nature 2011), tumor mutational burden (TMB), mutation count, and Spearman’s rank correlation analysis with genes of interest (Firehose Legacy) were determined.

##### Kaplan-Meier plotter analysis

The Kaplan-Meier Plotter ovarian cancer analysis (https://kmplot.com/analysis/index.php?p=service&cancer=ovar) (Lánczky and Győrffy, 2021) was used to examine the association of AREG with progression-free survival (PFS) and overall survival (OS) in stage III-IV, grade 3 serous ovarian cancer using either the lower or upper quartile as a cutoff.

##### Tumor immune dysfunction and exclusion

Tumor Immune Dysfunction and Exclusion (TIDE) (Jiang et al., 2018; Fu et al., 2020) query gene analysis was employed to examine AREG and cytotoxic T lymphocyte levels and T-cell dysfunction score/z-score of interaction between AREG and cytotoxic lymphocytes (CTLs) in a Cox proportional hazard model. TCGA ovarian cancer cohort was used by TIDE for these analyzes. As described detail in Jiang et al. (2018); briefly, an interaction test within the multivariate Cox-PH regression was applied to identify AREG genomic levels in association with the T cell dysfunction phenotype. Then the Cox-PH survival regression was employed to test how CTL levels interact with AREG in the tumor to affect overall survival outcomes. The linear model Hazard was solved (=*a*XCTL+*b*XV+*d*XCTLxV+C) using the Cox-PH regression, where the CTL level is estimated from the bulk-tumor expression average of cytotoxicity T cell markers (*CD8A*, *CD8B*, *GZMA*, *GZMB*, *PRF1*). The death hazard within the Cox-PH model was estimated via patient survival clinical outcome, the variable V is the expression level of the candidate gene in the test (in this case *AREG*). The T cell dysfunction score listed is defined as the Wald test z score, which represents the coefficient *d*, dived by its standard error. The *p*-value listed was adjusted using the Benjamini-Hochberg method.

## Results

### Bioinformatic analysis of AREG in ovarian cancer

**FIGURE 1:**
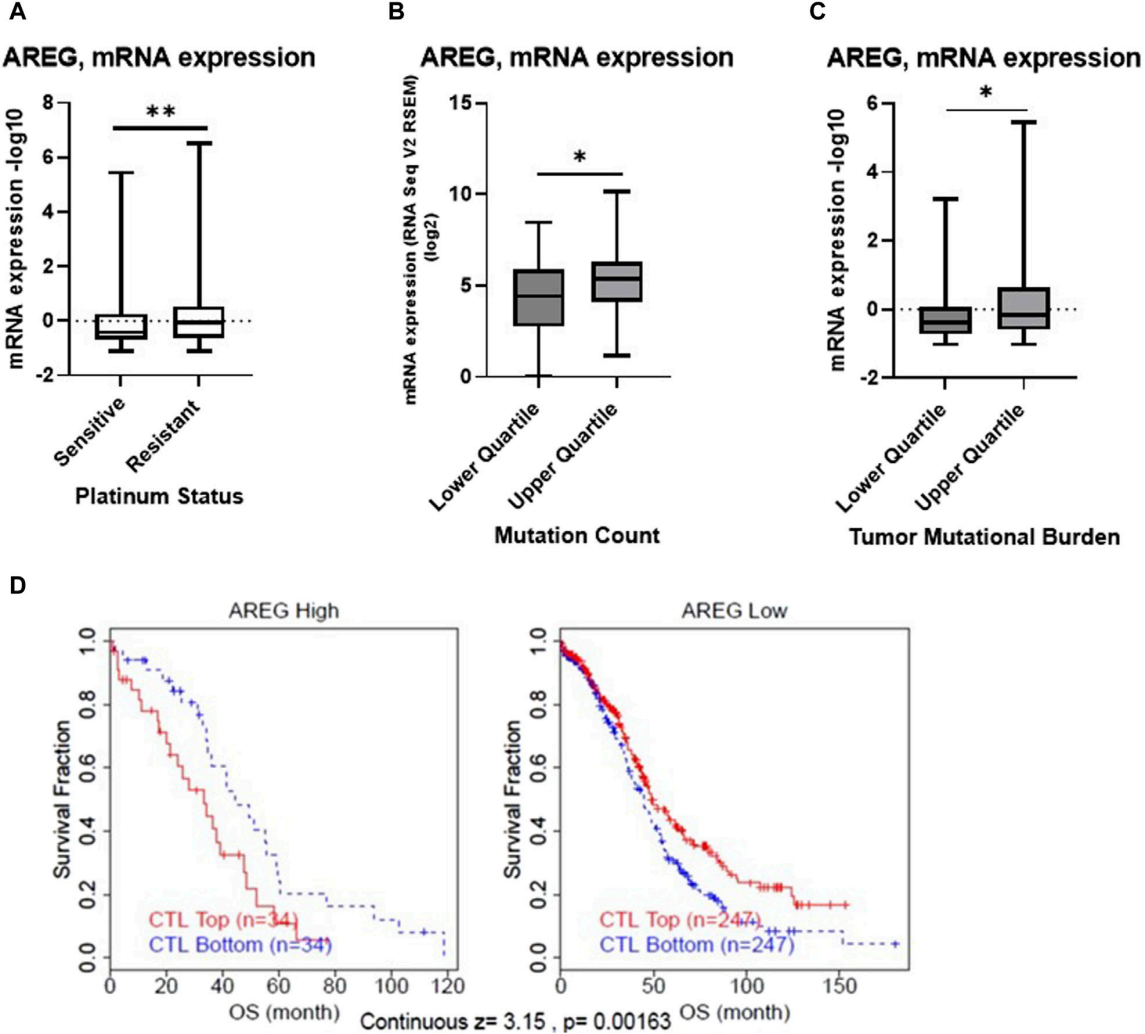
Bioinformatic analysis of AREG in ovarian cancer. TCGA Ovarian Cancer Nature 2011 cohort was employed to compare **(A)** mRNA expression of AREG in patients with platinum sensitive (*n* = 197) or resistant (*n* = 90) disease. The TCGA Ovarian Cancer Firehose Legacy was used to examine AREG mRNA expression stratified by upper and lower quartile **(B)** mutation count (*n* = 41, upper quartile, *n* = 52, lower quartile) and **(C)** tumor mutational burden (*n* = 79, for both upper and lower quartile). **(D)** TIDE cox proportional hazards model analysis of OS using TCGA ovarian cohort data demonstrating z-score of interaction effect between AREG and CTLs. *p < 0.05, **p < 0.005, as indicated. TCGA, The Cancer Genome Atlas; TIDE, Tumor Immune Dysfunction Exclusion; CTLs, Cytotoxic T lymphocytes; OS, overall survival.

Our past study revealed that AREG was significantly upregulated in HGSOC patient tumors following NACT exposure compared to matched pre-treatment diagnostic biopsy specimens (James et al., 2022). Therefore, using publicly available datasets we first sought to uncover *AREG*’s relationship to clinical outcomes in HGSOC. TCGA ovarian cancer cohort analysis revealed that *AREG* mRNA levels were significantly (*p* = 0.007) upregulated in patients defined as having a chemoresistant versus sensitive platinum status (Figure 1A). As approximately 80% of patients are defined as platinum sensitive, this small population of patients defined as chemoresistant exhibits an exceptionally poor survival of 6 months or less (Luvero et al., 2019). Interestingly, Kaplan Meier curve analysis of publicly available GSE and TCGA databases found no significant association between *AREG* expression and progression-free survival (PFS) or overall survival (OS; Supplementary Figure S1).

Further bioinformatic analysis revealed that despite being associated with chemoresistant disease, *AREG* mRNA levels were significantly (*p* < 0.05) higher in patients with a higher mutation count and tumor mutational burden (TMB), when stratified by quartile (Figures 1B, C). Moreover, Tumor Immune Dysfunction and Exclusion (TIDE) analysis revealed that higher levels of *AREG* were significantly (continuous z-score, 3.15, *p* = 0.00136) associated with a cytotoxic lymphocyte (CTL) dysfunction phenotype (Figure 1D). Overall, these results demonstrate that despite higher *AREG* levels detected in patient tumors with a higher TMB count, *AREG* was also associated with chemoresistant disease and T cell dysfunction.

### AREG exposure leads to tumor intrinsic immune changes that drive ovarian pathogenesis and immune evasion

**FIGURE 2:**
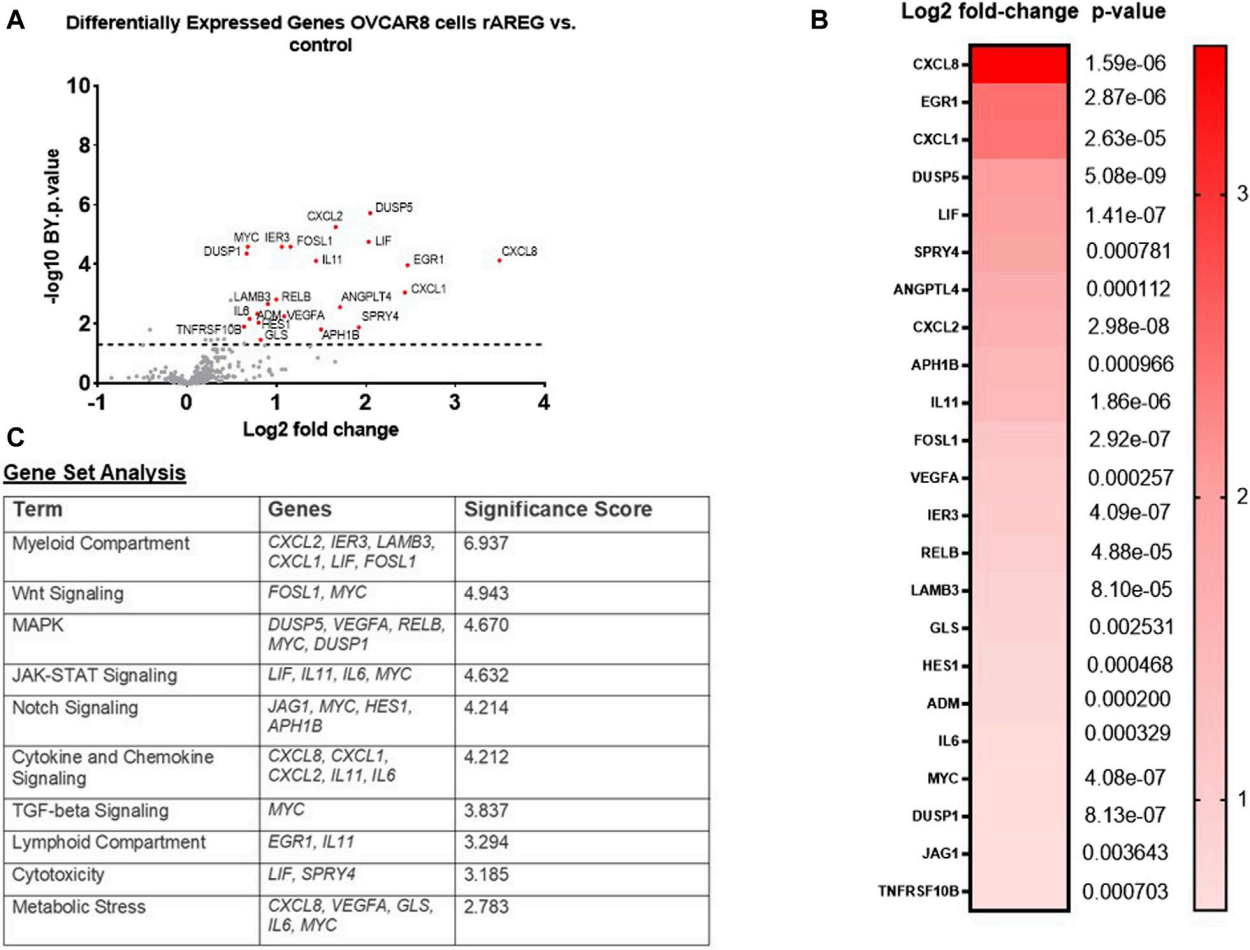
rAREG drives tumor intrinsic immune changes that promote immune evasion and ovarian pathogenesis. **(A)** Volcano plot demonstrating differential gene expression in OVCAR8 cells stimulated with 200 ng/mL of rAREG relative to BSA control, measured by NanoString Human PanCancer IO360. **(B)** Top differentially expressed genes in OVCAR8 rAREG treated cells relative to BSA control, with Benjamini-Hochberg adjusted *p*-values listed. **(C)** Gene set analysis with top pathway changes and significance scores listed in rAREG treated AREG cells relative to BSA control.

Next, in order to recapitulate the high levels of AREG that are seen in post-NACT treated HGSOC tumors, we stimulated the HGSOC cell lines OVCAR8 and PEA1 with 200 ng/mL recombinant AREG (rAREG) and respective controls for 2 h. Extracted RNA was subjected to NanoString IO 360 analysis with the goal of broadly capturing tumor intrinsic changes resulting from increased AREG exposure. Unexpectedly, we found no significant differences in PEA1 treated cells. However, in OVCAR8 cells, several genes were significantly upregulated relative to control, including *CXCL8* (3.49-fold, *p* = 1.59e-06), *EGR1* (2.47-fold, *p* = 2.87e-06), *CXCL1* (2.43-fold, *p* = 2.63e-05), *DUSP5* (2.05-fold, *p* = 5.08e-09), *LIF* (2.03-fold, *p* = 1.41e-07), *CXCL2* (1.66-fold, *p* = 2.98e-08), and IL-11 (1.44-fold, *p* = 1.86e-06; Figure 2A, B). Furthermore, gene set analysis revealed prominent changes in Wnt, MAPK, Notch, TGF-beta, JAK-STAT, and cytokine and chemokine signaling, as well as changes in cytotoxicity, metabolic stress and myeloid and lymphoid compartment (Figure 2C), showcasing that increased AREG leads to significant tumor intrinsic immune changes that can contribute to cell proliferation, migration, and angiogenesis, while simultaneously promoting tumor immune suppression.

**FIGURE 3:**
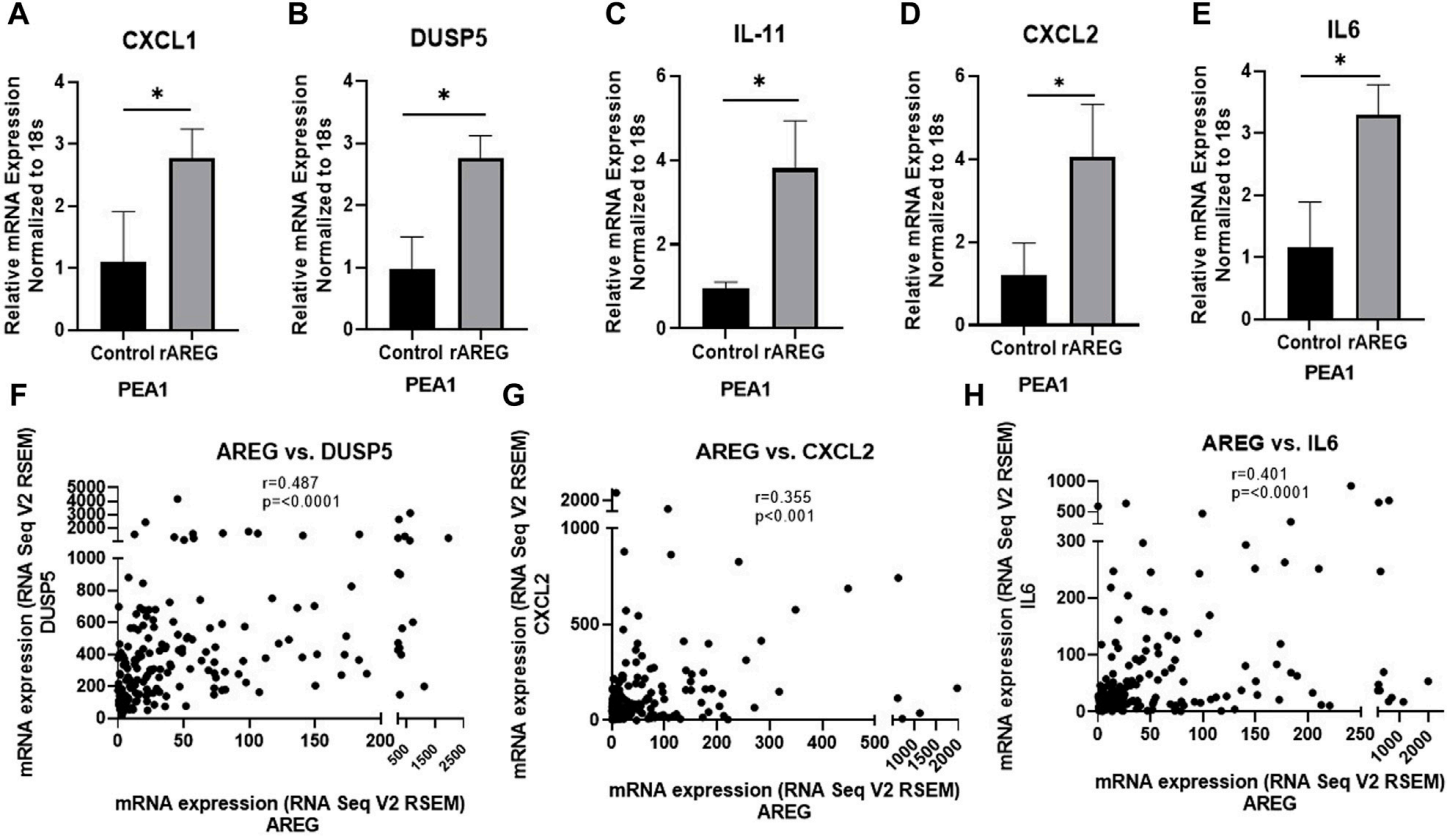
qPCR analysis of rAREG stimulated PEA1 cells. **(A)** *CXCL1*, **(B)** *DUSP5*, **(C)** *IL-11*, **(D)** *CXCL2*, and **(E)** *IL-6* mRNA levels in PEA1 cells stimulated with 200 ng/mL rAREG for 1 h and analyzed via qPCR. Spearman Rank Correlation analysis of mRNA expression (RNA Seq V2 RSEM) of *AREG* with **(F)** *DUSP5*, **(G)** *CXCL2*, and **(H)** *IL-6* using TCGA-OV Firehose Legacy cohort (*n* = 307). Error bars represent standard deviation of ≥ 3 biological replicates. *p < 0.05 as indicated. TCGA, The Cancer Genome Atlas.

Following our NanoString analysis, we re-treated PEA1 cells with 200 ng/mL of rAREG and collected RNA at an earlier 1h timepoint. We performed quantitative PCR (qPCR) with the goal of examining levels of differentially expressed genes (DEGs) identified in OVCAR8 cells. We found that mRNA levels of *CXCL1* (2.53-fold, *p* = 0.036), *DUSP5* (2.82-fold, *p* = 0.008), *IL-11* (4.01-fold, *p* = 0.012), *CXCL2* (3.36-fold, *p* = 0.029), and *IL6* (2.84-fold, *p* = 0.013), were all significantly increased following 1h rAREG exposure (Figures 3A–E), with no significant changes at 2 h stimulation (Supplementary Figure S2), confirming what we previously observed in our NanoString analysis. The discrepancies in OVCAR8 and PEA1 could potentially be explained by the fact that it is known that OVCAR8 cells harbor *Erbb2* and *KRAS* mutations (Mei et al., 2021), which could lead to differential rAREG effects. Finally, we observed that the DEGs *DUSP5* (r = 0.487, *p* < 0.0001), *CXCL2* (r = 0.355, *p* < 0.0001), and *IL-6* (r = 0.401, *p* < 0.0001) were amongst some of the top correlative genes with *AREG* in the TCGA ovarian cancer cohort (Figures 3F–H), adding a further degree of clinical relevance to our NanoString analysis.

### AREG promotes upregulation of downstream EGFR cell growth pathways

**FIGURE 4:**
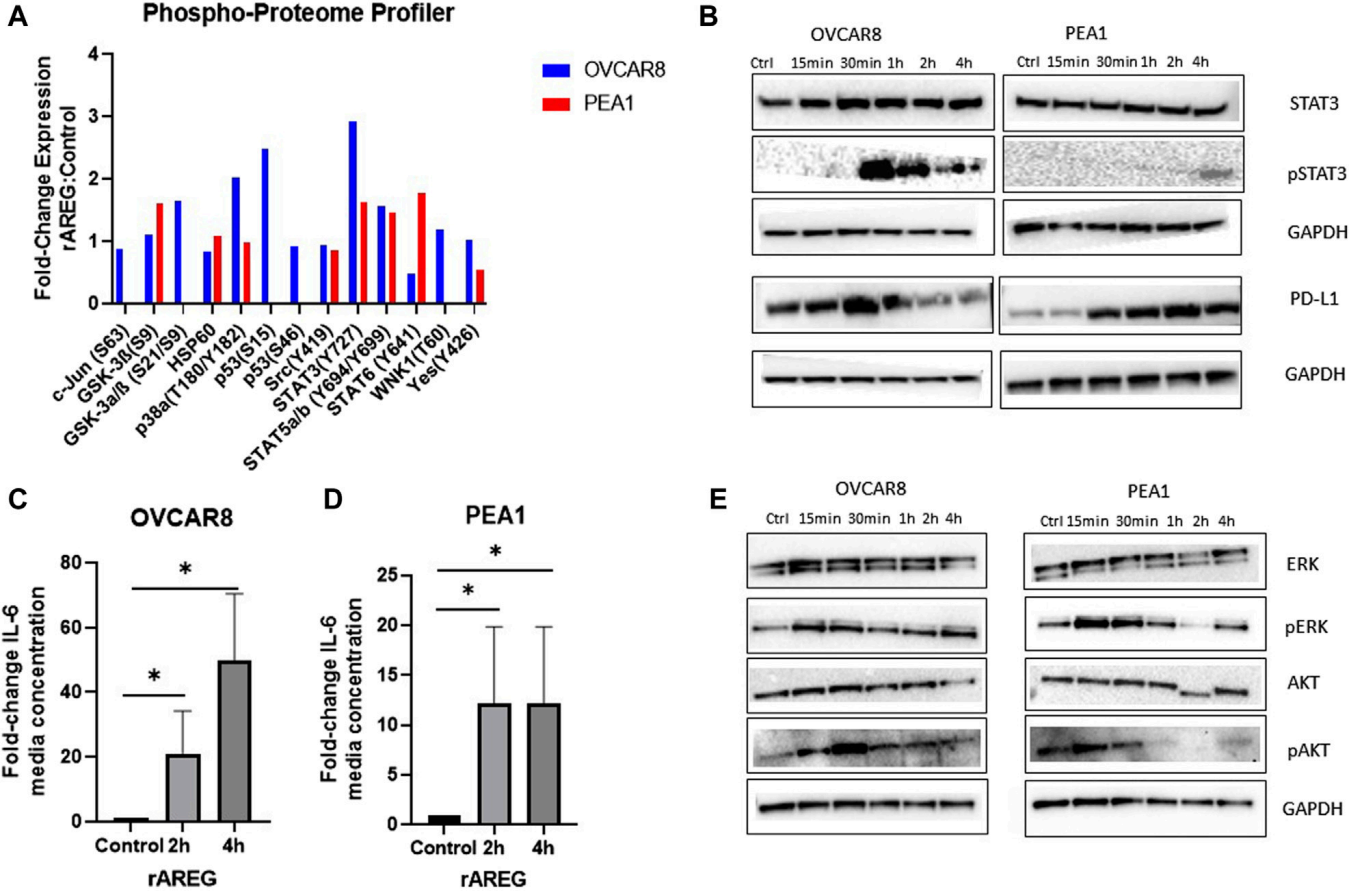
AREG exposure leads to prominent upregulation of STAT3 signaling in HGSOC cells. **(A)** Phospho-proteome profiler analysis of fold-change expression of phosphor-proteins in rAREG (200 ng/mL) treated OVCAR8 and PEA1 cells relative to BSA control. **(B)** Western blot analysis of rAREG (200 ng/mL) exposed OVCAR8WT and PEA1 cells of STAT3, pSTAT3, PD-L1, with respective GADPH loading controls at various indicated timepoints. ELISA levels of IL-6 in conditioned media of **(C)** OVCAR8 and **(D)** PEA1 cells following 200 ng/mL of rAREG exposure at 2 and 4 h or BSA control. **(E)** Western blot analysis of downstream EGFR cell growth pathways p-ERK/ERK and pAKT/AKT with respective GAPDH loading controls in OVCAR8 and PEA1 cells following rAREG treatment at various indicated timepoints.*p < 0.05, as indicated. ELISA, enzyme-linked immunosorbent assay.

As the EGFR pathway is upstream of numerous cancer cell growth pathways (Wee and Wang, 2017), we employed a commercially available proteome profiler array to unbiasedly uncover notable signaling changes in HGSOC cells following rAREG exposure. Interestingly, we found that STAT3 expression was upregulated 2.93-fold in OVCAR8 and 1.63-fold in PEA1 cells after only 15 min of exposure (Figure 4A). Western blot analysis was employed to validate findings and compare phospho-STAT3 (p-STAT3) levels at multiple timepoints, which revealed the highest upregulation of p-STAT3 at 1 h and 4 h in OVCAR8 and PEA1 cells, respectively (Figure 4B). Moreover, we found that programmed-death ligand 1 (PD-L1), a major immune target downstream of the STAT3 pathway (Zerdes et al., 2019; Zou et al., 2020), was also increased strikingly starting at 30 min following rAREG treatment in both OVCAR8 and PEA1 cells (Figure 4B).

To further examine AREG’s influence on the STAT3 pathway, we evaluated secreted levels of the pro-inflammatory and major STAT3-associated cytokine, IL-6, in media from rAREG stimulated HGSOC cells. At both 2 h and 4 h time points following rAREG exposure, IL-6 levels in conditioned media were 21.1-fold and 49.6-fold higher in OVCAR8 cells, respectively and 12.2-fold higher at both 2 h and 4 h post-rAREG exposure timepoints in PEA1 cells (IL-6 levels reached or exceeded the upper limit of detection at 500 pg/mL; Figures 4C, D). As IL-6 possesses the unique ability to induce STAT3 target genes, which in turn produce multifaceted downstream effects that drive tumor cell growth, angiogenesis, invasion, metastasis, and immunosuppression (Wang and Sun, 2014; Čokić et al., 2015; Johnson et al., 2018), our results highlight AREG’s indirect pro-tumorigenic effects through IL-6 stimulation. In addition, we treated OVCAR8 and PEA1 cells with ruxolitinib, a small molecule JAK/STAT3 inhibitor (Han et al., 2018), which resulted in unaltered AREG levels (Supplementary Figure S3), suggesting that STAT3 does not have a bidirectional influence on AREG in HGSOC.

Finally, Western blot analysis revealed that rAREG exposure led to activation of additional tumor cell growth pathways downstream of EGFR, illustrated by increased p-ERK and p-AKT levels starting at 15 min of exposure in both OVCAR8 and PEA1 cells (Figure 4E). Taken together, these results showcase that AREG greatly contributes to the activation of numerous cell growth pathways in HGSOC, with predominant effects on STAT3 and its associated targets.

### AREG reduces cytotoxic immune response *in vitro*

**FIGURE 5:**
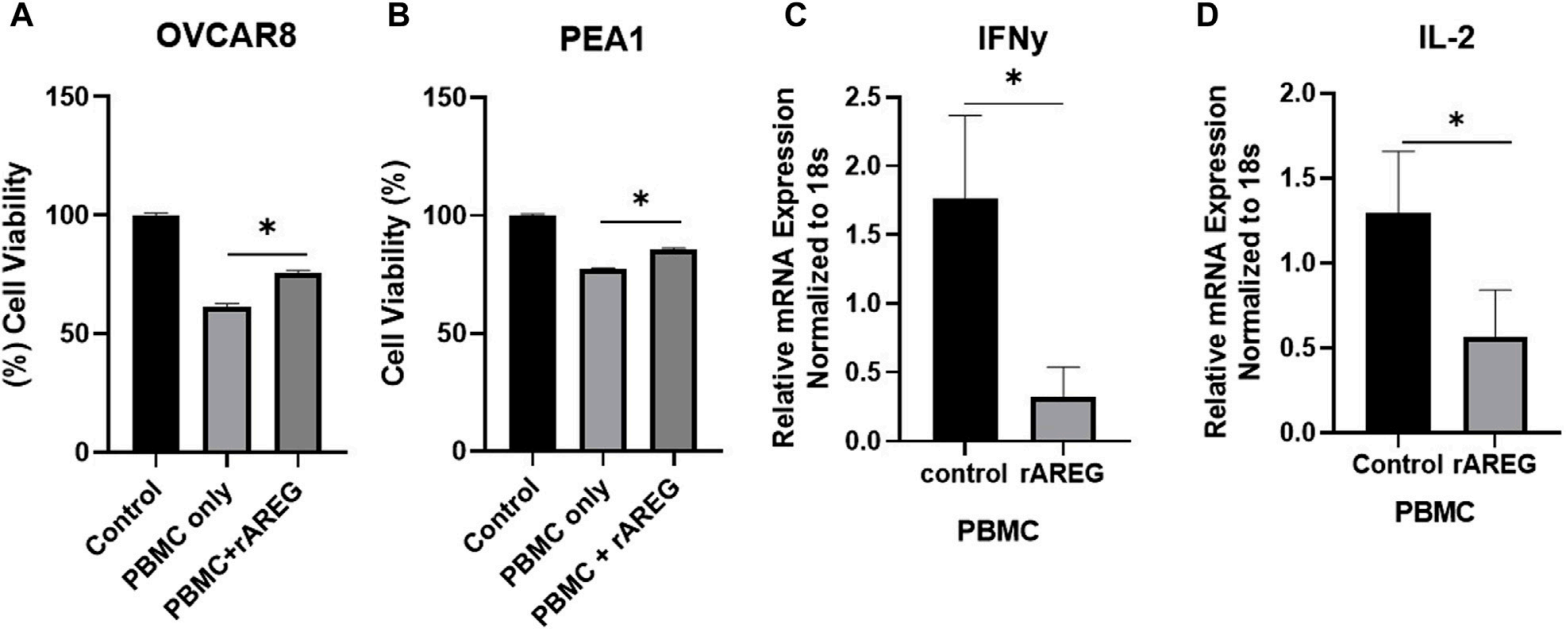
AREG compromises PBMC cytotoxicity. Cell viability analysis **(A)** OVCAR8 and **(B)** PEA1 cells following 24-h co-cultured with PBMCs+ BSA control or PBMCs+ 200 ng/mL of rAREG. qPCR analysis of **(C)** *IFNy* and **(D)** IL-2 in PBMCs treated with BSA control, or rAREG for 2 h. Error bars represent standard deviation of ≥ 3 biological replicates. *p < 0.05, as indicated, PBMCs, peripheral blood mononuclear cells.

As we previously found that AREG leads to tumor intrinsic immune changes that drive ovarian pathogenesis and promote immune evasion, we sought to evaluate if increased AREG exposure affects cytotoxic immune responses. To investigate this phenomenon, we co-cultured OVCAR8 and PEA1 cells with peripheral blood mononuclear cells (PBMCs) that were stimulated with or without rAREG for 24-h. We observed significantly (*p* = 0.001) reduced viability of 38.8% in OVCAR8 cells stimulated with PBMCs + BSA control compared to a 24.2% reduction in viability in cells stimulated with PBMCs + rAREG (Figure 5A). Similarly, PEA1 cells co-cultured with PBMCs + BSA demonstrated a 22.6% reduction in viability, compared to 14.4% with PBMCs + rAREG (*p* = 0.007; Figure 5B). Furthermore, we stimulated PBMCs alone with rAREG and performed qPCR analysis, which revealed a significant (*p* < 0.05) decrease in both *IL-2* and *IFNγ* (Figures 5C, D), in addition to a trend toward reduced *GZMB* levels (Supplementary Figure S4). Collectively, these studies show that increased AREG dampens PBMCs’ ability to promote tumor cell death potentially through the reduction of cytokines crucially responsible for carrying out cytotoxic immune responses.

### *In vivo* AREG exposure predominantly drives immunosuppresive adaptations within the OTIME

**FIGURE 6:**
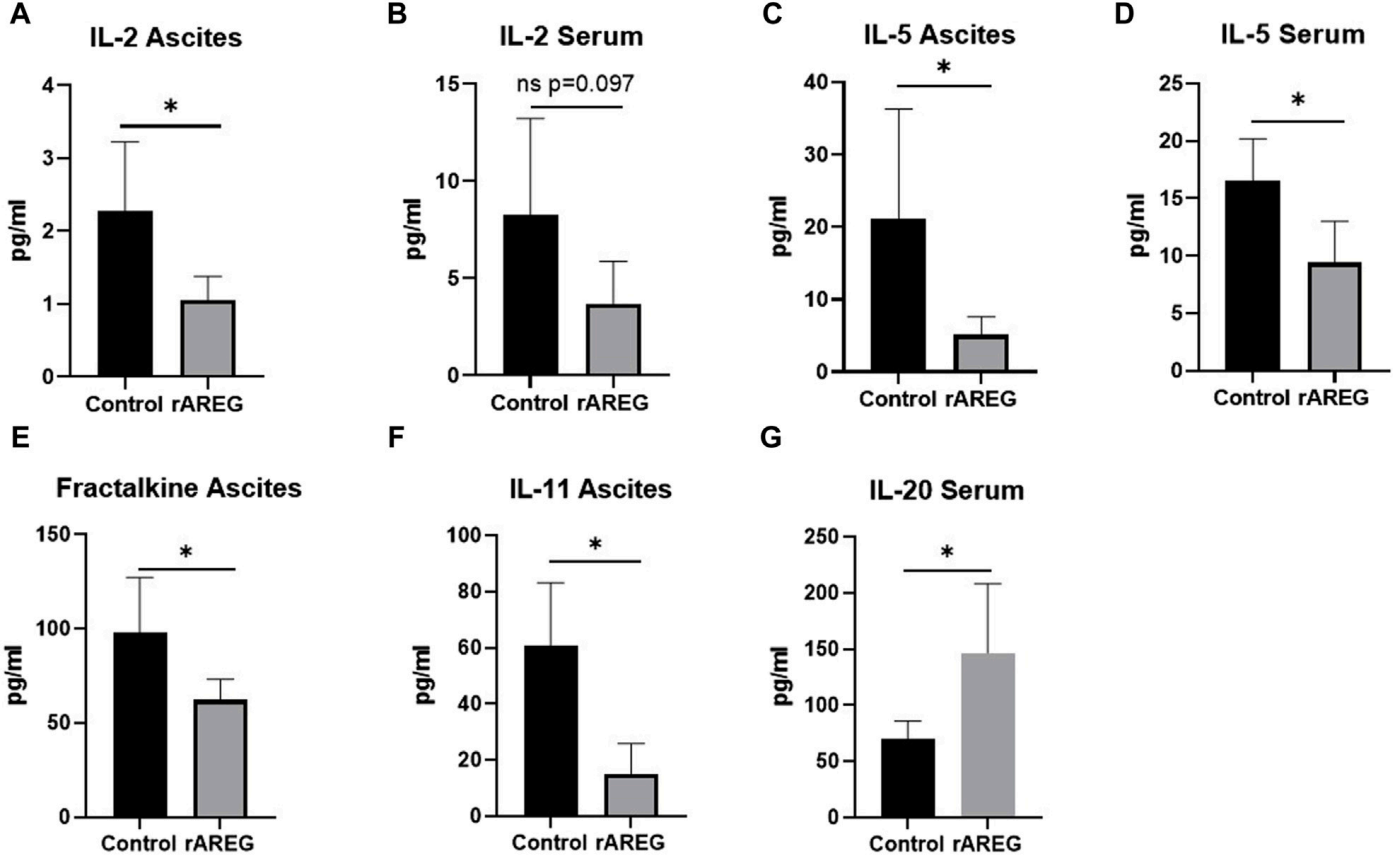
Multiplex cytokine and chemokine analysis of C57BL/6 ID8p53^−/−^ mouse ascites and serum following *in vivo* rAREG exposure. Concentrations of IL-2 in **(A)** serum and **(B)** ascites of mice treated with saline control (*n* = 5) or 400 ug/kg of rAREG (*n* = 5). IL-5 levels in the ascites **(C,D)** serum in mice exposed to saline control or rAREG. **(E)** Fractalkine ascites, **(F)** IL-11 ascites, and **(G)** IL-20 serum levels in saline and rAREG treated mice. *p < 0.05 as indicated.

In order to characterize the effect of AREG on the ovarian tumor immune microenvironment, we carried out an immunocompetent *in vivo* study using an ID8p53^−/−^ C57BL/6 model in which mice were treated with 400 μg/kg of rAREG or a saline control. Ascites and serum obtained post-euthanasia were submitted for multiplex cytokine and chemokine analysis which revealed a significant (*p* = 0.026) reduction of IL-2 levels in ascites of mice treated with rAREG compared to saline control mice (Figure 6A). A similar reduction in IL-2 levels was seen in rAREG treated mouse serum, however this did not reach significance (*p* = 0.097) (Figure 6B). This result corroborates our *in vitro* findings that rAREG exposure leads to reduced *IL-2* mRNA levels in PBMCs. Interestingly, we also observed that mice treated with rAREG had significantly (*p* < 0.05) reduced ascites and serum levels of IL-5, a pro-inflammatory cytokine that is primarily responsible for eosinophil production (Han et al., 2011) (Figures 6C, D). Furthermore, a significant (*p* = 0.034) reduction of Fractalkine, also known as CX3CL1 was observed in rAREG treated mouse ascites, which has been found to be a key mediator in of cytotoxic T cell immunity and associated with improved prognosis in numerous cancer subtypes (Conroy and Lysaght, 2020) (Figure 6E). In addition, a significant (*p* = 0.004) reduction in IL-11, an IL-6 associated cytokine (Zhao et al., 2018) was also was also observed (Figure 6F), which we previously observed to be upregulated in a tumor-intrinsic setting (Figures 2A, 3C). Finally, there was a significant (*p* = 0.028) increase in IL-20 (Figure 6G), a potent inflammatory cytokine that is classically associated with psoriasis and rheumatoid arthritis but has also been shown to promote tumorigenesis through promoting cellular proliferation and migration (Hsu et al., 2012a; Hsu et al., 2012b; Chiu et al., 2017; Lu et al., 2020). A complete list of all changes in cytokines and chemokines profiled can be seen in Supplementary Tables S1, S2.

**FIGURE 7:**
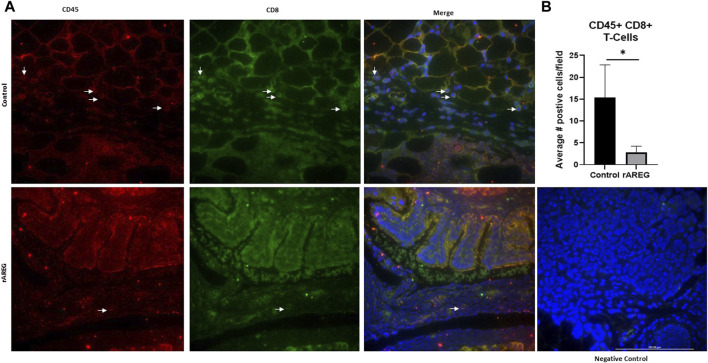
AREG exposure *in vivo* leads to a reduction in intratumoral cytotoxic CD8^+^ T cells. **(A)** Representative images and associated **(B)** Fluorescent Immunohistochemistry analysis of intratumoral CD8^+^ T cells levels denoted by the number of positive cells per field in saline control (*n* = 3) and 400 μg/kg rAREG exposed (*n* = 4) mice.*p < 0.05, as indicated.

**FIGURE 8:**
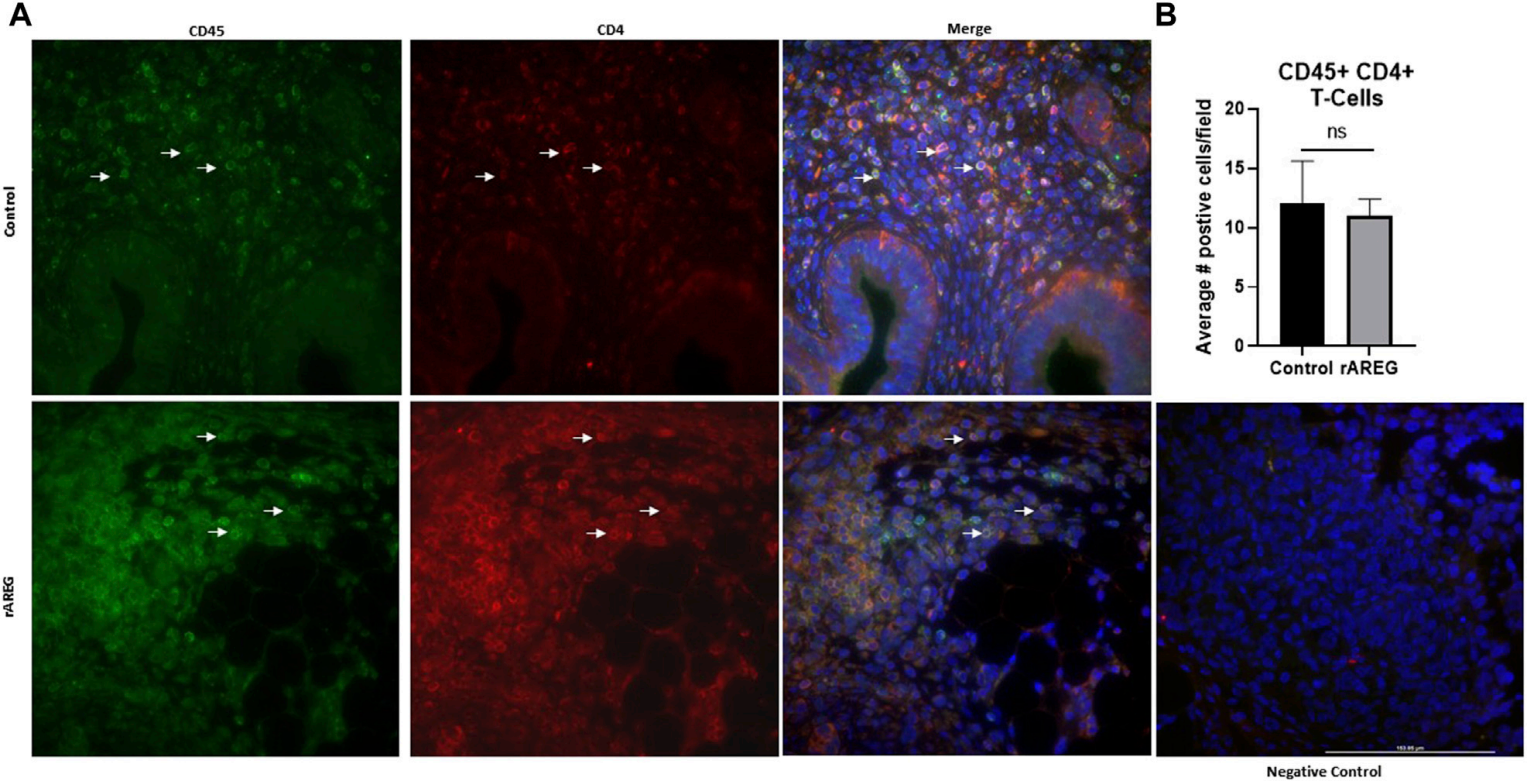
Intratumoral analysis of CD4^+^ T cells following *in vivo* rAREG exposure. **(A)** Representative images and associated **(B)** Fluorescent Immunohistochemistry analysis of intratumoral CD4^+^ T cells (number of positive cells per field) in saline control (*n* = 3) and 400 μg/kg rAREG exposed (*n* = 4) mice. ns, non-significant.

**FIGURE 9:**
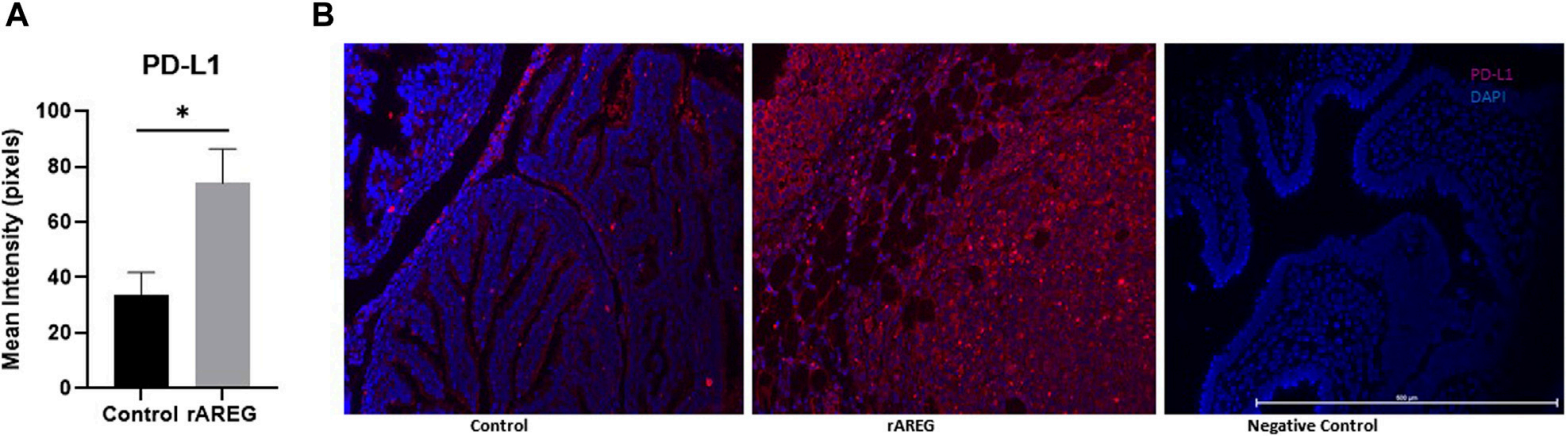
AREG exposure leads to an upregulation of intratumoral PD-L1 expression. Fluorescent Immunohistochemistry analysis of **(A)** of PD-L1 in saline control (*n* = 3) and 400 μg/kg rAREG exposed (*n* = 3) mice, demonstrated by mean intensity (pixels) with **(B)** representative images. *p < 0.05, as indicated.

In addition to evaluating circulating changes within the OTIME, we further observed a significant (*p* = 0.0212) reduction in the average number of intratumoral CD8^+^ T cells, with an average of five positive CD8^+^ T cells per field in saline tumors compared to one positive CD8^+^ T cell per field in mice exposed to rAREG (Figures 7A, B). Conversely, we observed no significant changes in CD4^+^ T cell populations (Figure 8). Finally, these tumors were also stained for PD-L1, which revealed significantly (*p* = 0.009) higher mean intensity levels of PD-L1 in tumors treated with rAREG compared to saline control (Figures 9A, B), recapitulating our results in HGSOC cell lines.

### Combinatorial AREG inhibition and carboplatin promotes synergistic HGSOC cell death

**FIGURE 10:**
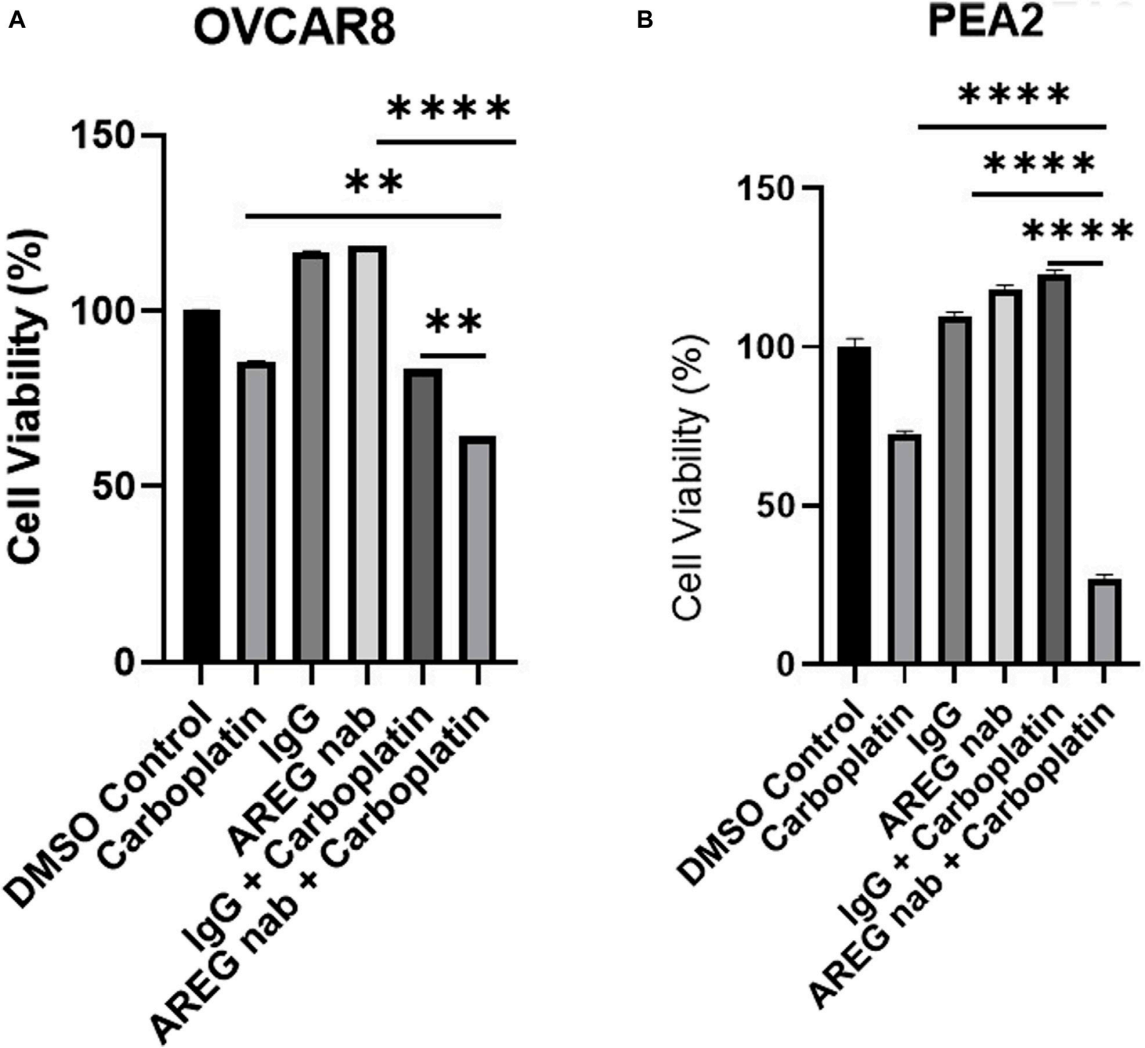
Targeting AREG in combination with carboplatin synergistically reduces HGSOC cell viability. Cell viability analysis of **(A)** OVCAR8 and **(B)** PEA2 cells pre-treated with carboplatin (300 μM for OVCAR8 cells, 400 μm for PEA2 cells) for 24 h and then stimulated with 30 μM of AREG nab or corresponding IgG control for an additional 48 h. Error bars represent standard deviation of ≥ 3 biological replicates. **p < 0.005, ***p < 0.0005, ****p < 0.00005, as indicated. AREG nab, AREG neutralizing antibody.

Finally, we have targeted AREG *in vitro* using an AREG neutralizing antibody (nab) in combination with carboplatin. HGSOC cell lines OVCAR8 and PEA2 (the chemoresistant counterpart to PEA1), were employed for this experiment. Both cell lines were pre-treated with carboplatin for 24 h and then treated with either an IgG control or AREG nab for 48 h. In both cell lines, it was observed that co-treatment with carboplatin and an AREG nab led to a significant (*p* < 0.005) reduction in cell viability compared to either carboplatin or AREG nab treatment alone, with the most striking reduction in chemoresistant PEA2 cells where combinatorial treatment produced a 73% reduction in viability compared to DMSO control (Figures 10A, B). While these cell viability assays were performed in an immune devoid context, it will be pertinent to validate these findings using an immunocompetent *in vivo* model.

## Discussion

The goal of this study was to characterize how increased AREG levels that are detected clinically in post-frontline chemotherapy exposed HGSOC patient tumors impacts the OTIME. This investigation revealed that enhanced AREG exposure produced multifaceted effects within the OTIME that collectively drive tumor immune evasion. While it has been previously reported that AREG is overexpressed in ovarian cancer (Lindzen et al., 2021) and is associated with advanced stage disease (Tung et al., 2017), using bioinformatic analyses we failed to observe an association between *AREG* expression and patient survival. Previously performed *in vitro* studies led by Tung et al. described AREG’s role in ovarian cancer chemoresistance through the promotion of cancer stemness and drug resistance mediated by the EGFR/ERK pathway (Tung et al., 2017). Similarly, the role of AREG in chemoresistance has been described in other cancer subtypes (Yoshida et al., 2012; Hsieh et al., 2019; Xu et al., 2019; Huang et al., 2020). Our analysis of publicly available datasets revealed AREG’s upregulation in chemoresistant ovarian cancer patients, further strengthening our previous observation of AREG’s significant upregulation in HGSOC patient tumors following exposure to frontline carboplatin and paclitaxel (James et al., 2022).

In this present investigation we first sought to specifically uncover how elevated AREG expression impacts tumor intrinsic immune changes. Interestingly, we found through our NanoString analysis that exposure of HGSOC cells to AREG led to an upregulation in genes related tumor cell growth, angiogenesis, and immune evasion. Most notably, we saw significant changes in angiogenic factors *CXCL8* and *VEGFA* (Marjon et al., 2004), as well as prominent changes in *CXCL1* and *CXCL2*, two chemokines known to contribute to chemoresistance via the recruitment of myeloid derived suppressor cells (MDSCs) (Ozga et al., 2021) and known to be associated with ovarian tumorigenesis (Zhang et al., 2021; Korbecki et al., 2023). Finally, pathway analysis revealed substantial upregulation of genes associated with STAT3 and MAPK/ERK signaling in HGSOC cells. Increased STAT3 and MAPK/ERK activation was confirmed via Western blot, while simultaneously detecting increased AKT pathway activation following rAREG exposure. Plausibly, it can be inferred that the observed increases in multiple cell growth pathways following rAREG treatment can be attributed to AREG’s unique binding to its receptor EGFR. AREG’s characterization as a low affinity EGFR ligand is due to a single amino acid mutation in its receptor binding domain which produces an unstable interaction with the EGFR receptor and consequential failure of EGFR internalization and enhanced downstream signaling. In contrast, when a high affinity ligand such as EGF binds to EGFR, this action promotes rapid internalization and associated negative feedback signaling loops from downstream cell growth pathways (Zaiss et al., 2015). While one limitation of this study is that we did not we did not confirm that AREG’s mechanism of action is indeed through EGFR, this will be imperative to investigate in further studies.

Our finding that AREG robustly activates the STAT3 pathway is particularly noteworthy given STAT3’s widespread effects on immunosuppression, cell proliferation, angiogenesis, and metastasis (Zou et al., 2020). Moreover, there has been vested clinical interest in targeting the STAT3 pathway with small molecule inhibitors (Song et al., 2023). Intriguingly, ruxolitinib, a JAK/STAT inhibitor, which is known to inhibit pSTAT3 in ovarian cancer cells (Han et al., 2018), was recently evaluated in a phase I/II clinical trial in combination with frontline carboplatin and paclitaxel chemotherapy (NRG-GY007, NCT02713386) (Landen et al., 2022). Despite the fact that the addition of ruxolitinib was narrowly insignificant, this study demonstrated feasibility and acceptable toxicity and has opened the door for additional combination approaches including ruxolitinib in ovarian cancer in the frontline setting. As this present study has shown that increased AREG levels promote STAT3 signaling activation, targeting AREG could conceivably lead to a reduction in STAT3 activation concomitantly with other growth signaling pathways, and potentially reduce immunosuppression within the OTIME. While our present investigation did not evaluate this hypothesis, it will be pertinent to examine how inhibiting AREG affects downstream cell growth pathways such as STAT3.

To the best of our knowledge, this study is the first to show that AREG promotes upregulation of intratumoral PD-L1 levels in HGSOC, as there been only one study in prostate cancer that previously demonstrated that paracrine AREG induces PD-L1 activity (Xu et al., 2019). Binding of programmed cell death 1 (PD-1) to PD-L1 has been established as one of the critical ways in which tumor cells become able to evade immune surveillance (Pardoll, 2012). Immunotherapies targeting the PD-1/PD-L1 axis have revolutionized the field of oncology, however, monotherapy response rates to PD-1/PD-L1 inhibitors have demonstrated low overall response rates (ORRs) in HGSOC. Despite this fact, it is well known that PD-L1 is highly expressed in ovarian tumors (Alwosaibai et al., 2023) and that anti-tumor immune responses are detected in ovarian tumors (Preston et al., 2011). Therefore, it has been suggested that due to the highly immunosuppressive nature of the OTIME (James et al., 2020), more than one immunotherapeutic approach may be necessary to effectively combat this immunosuppression. Hence, our finding that AREG directly contributes to HGSOC immunosuppression through upregulating PD-L1 expression indicates that targeting AREG in combination with PD-1/PD-L1 blockade could potential improve HGSOC response rates to clinically available PD-1 based immunotherapies. Future pre-clinical studies to evaluate this hypothesis are necessary.

Using *in vitro* and *in vivo* models, this investigation has established that AREG compromises cytotoxic immune responses in HGSOC. It has been widely reported that AREG has a role in promoting immunosuppression within the context of classical immunity. AREG is known to be expressed by Tregs and directly fosters Treg function through the secretion of exosomes that transfer immunosuppressive micro-RNAs to effector T cells (Zaiss et al., 2015). In addition, it is known that AREG possesses the ability to downregulate costimulatory B7 molecules, enhancing cytotoxic T cell death (Dreschers et al., 2023). While AREG’s role in classical immunity has been well defined, its specific function in the context of tumor immunology has been comparatively understudied. We have shown for the first time in HGSOC that elevated AREG exposure *in vivo* leads to a reduction in intratumoral CD8^+^ T cells. Interestingly, a study by Yuan et al. (2015) found that Tregs co-cultured with CD8^+^ T cells in the presence of AREG led to a reduction in CD8^+^ T cell activation markers such as IFNy. While a limitation of our study is that we did not specifically isolate these T cell subsets, we similarly observed a reduction in cytotoxic responses with significant reductions in IFNy and IL-2 expression in PBMCs cultured with rAREG. Yuan et al. further discovered that EGFR was not expressed by either intratumoral or splenic CD8^+^ T cells and that blocking AREG inhibited Treg activation specifically, leading the group to postulate that AREG does not likely impact CD8^+^ T cells directly, but through influencing Treg function (Yuan et al., 2015). Moreover, studies in melanoma, as well as gastric and lung cancer have similarly observed that AREG leads to immunosuppression through the regulation of Treg function (Wang et al., 2016; Green et al., 2017; Sun et al., 2023). While our present study only identified the reduction of cytotoxic responses, it will be pertinent to also examine how AREG affects Treg function as well as other pertinent immune cell subsets within the OTIME. These future studies will be critical in order to understand how AREG mechanistically compromises cytotoxic immune responses in HGSOC.

Out of an extensive panel of chemokines and cytokines, IL-5 was found to be significantly downregulated by *in vivo* rAREG exposure in both ascites and serum. IL-5 is an essential cytokine required for eosinophil development, and like AREG functions as a Th2 cytokine (Dent et al., 1990; Morimoto et al., 2018). Several studies have demonstrated that IL-5 and eosinophils are vital to the production of anti-tumor immune response (Ikutani et al., 2012; Blomberg et al., 2023; Jacenik et al., 2023). Hence, the ability of AREG to downregulate IL-5 may potentially contribute to the suppressive OTIME and the muted response to immunotherapies that is seen clinically in HGSOC. While this to the best of our knowledge is the first study to identify the relationship between AREG and IL-5 in the context of tumor immunology, it is known that AREG is expressed by human eosinophils in response to IL-5 exposure (Matsumoto et al., 2009). Moreover, connections between IL-5 and AREG have been reported in the severe asthma and lung fibrosis (Morimoto et al., 2018; Bagnasco et al., 2020). Future mechanistic examination examining how IL-5 and AREG interact in the context OTIME are warranted.

Similar to our *in vivo* analysis, which found a decrease of IL-2 expression in PBMCs with rAREG exposure, we also saw a marked reduction of IL-2 in ascites from mice exposed to rAREG. Interestingly, recombinant IL-2 has been a long-standing immunotherapy, with the goal of eliciting anti-tumoral immune responses. However, there have been major limitations associated with this therapy due to systemic toxicity, which has prevented many cancer patients from benefiting from IL-2 treatment (Briukhovetska et al., 2021). Recently, a Phase 1/2 trial was initiated to analyze the safety and efficacy of encapsulated IL-2 nanoparticles administered intraperitoneally (AVB-001; NCT05538624), specifically in a cohort of recurrent HGSOC patients, with the goal of maximizing cytotoxic immune activation and decreasing toxicity through local peritoneal administration. Overall, our data shows that AREG treatment leads to the pronounced downregulation of a vital pro-inflammatory, clinically relevant HGSOC cytokine IL-2.

We have shown that even in an immune devoid context, targeting AREG in combination with HGSOC standard of care chemotherapy synergistically promotes HGSOC cell death. Two prior studies have targeted AREG in ovarian cancer mouse models. The first, a study by Lindzen et al. (2021) found that an AREG is significantly abundant in ovarian cancer patient ascites and that treatment with an AREG blocking antibody led to prolonged survival in an immunocompetent *in vivo* wildtype p53 HGSOC model. The authors theorized that this efficacy is attributed to the presumed binding of wildtype p53 to AREG’s promoter which in turn leads to EGFR activation that can be blocked by an AREG monoclonal antibody (Lindzen et al., 2021). However, given that p53 is mutated in over 96% of all HGSOC (Oien et al., 2016), this finding is clinically relevant to a minute subset of HGSOC patients. The second study by Carvalho et al. (2016) found that an AREG neutralizing antibody as a single agent and in combination with cisplatin led to a synergistic reduction in tumor burden. Although promising, this study was performed in a nude xenograft model and therefore cannot inform consequences of AREG inhibition on the OTIME. In a prostate cancer model, Xu et al. (2019) tested combinatorial AREG blockade with chemotherapy, which demonstrated superior anti-tumor efficacy, even compared to co-treatment with the EGFR mab cetuximab and chemotherapy. Fascinatingly, this finding suggests that EGFR may not be AREG’s sole surface receptor within the tumor immune microenvironment (Xu et al., 2019). In the future, it will be necessary to examine the combinatorial efficacy of AREG and chemotherapy regimens in an immunocompetent HGSOC *in vivo* model, in order to determine if this strategy leads to reduced tumor burden and rescues the rAREG-induced diminished cytotoxic immune responses that we have seen in this present study. Furthermore, as was evaluated by Xu et al. (2019), it would be worthwhile to compare the efficacy of AREG neutralization with EGFR blockade in order to further elucidate AREG’s mechanism of action within the OTIME.

In conclusion, this study demonstrates that AREG promotes immunomodulation within the OTIME and leads to the reduction of cytotoxic responses, indicating its putative role as a novel HGSOC immune target. In addition, AREG’s function in promoting chemoresistance and PD-L1 immune dysfunction provides strong rationale for combinatorial approaches with HGSOC standard of care chemotherapy and PD-1 based immunotherapy. Future pre-clinical studies testing these new immune modulating regimens will be informative to a patient population that has yet to respond meaningfully to clinically available immunotherapies.

## Data Availability

The datasets presented in this study can be found in online repositories. The names of the repository/repositories and accession number(s) can be found below: https://www.ncbi.nlm.nih.gov/geo/, GSE252495.

## Supplementary material

SUPPLEMENTARY FIGURE S1 AREG is not associated with HGSOC survival outcomes. Kaplan-Meier Plotter analysis using TCGA analysis and Gene Expression Omnibus Series data in patients with stage 3 and 4 ovarian cancer, demonstrating AREG’s association with PFS stratified by **(A)** lower quartile and **(B)** upper quartile AREG levels. AREG’s association with OS stratified by **(C)** lower quartile and **(D)** upper quartile AREG levels. TCGA, The Cancer Genome Atlas; PFS, progression-free survival; OS, overall-survival.

SUPPLEMENTARY FIGURE S2 PEA1 qPCR analysis at 2 h rAREG exposure timepoint. qPCR analysis CXCL1 **(B)** DUSP5 **(C)** IL-11 **(D)** CXCL2 **(E)** IL-6 of PEA1 cells stimulated with 200 ng/ml of rAREG or BSA control for 1 h. ns, non-significant.

SUPPLEMENTARY FIGURE S3 Small molecule JAK/STAT inhibition does not affect AREG levels in HGSOC cells. Western blot analysis of AREG levels and corresponding GAPDH loading control in PEA1 and OVCAR8 cells following 10 μm of ruxolitinib treatment for 48 h.

SUPPLEMENTARY FIGURE S4 qPCR analysis of GRZB in PBMCs. ns, non-significant; PBMC, peripheral blood mononuclear cell.
