# Supplementary figures and images for: Enhanced amphiregulin exposure promotes modulation of the high grade serous ovarian cancer tumor immune microenvironment
Source: Front Pharmacol. 2024 May 20;15:1375421. doi: 10.3389/fphar.2024.1375421 (PMC11144882; doi:10.3389/fphar.2024.1375421)

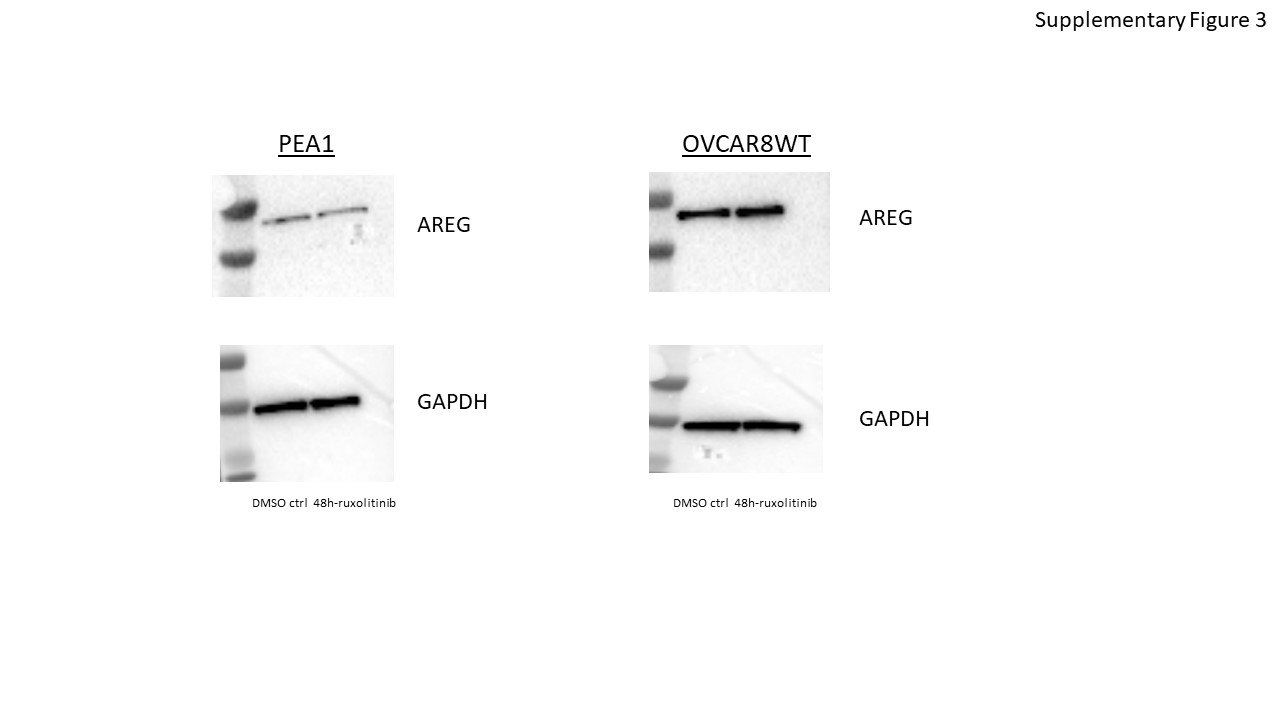

Supplement: Supplementary file 1 [file Image3.JPEG]

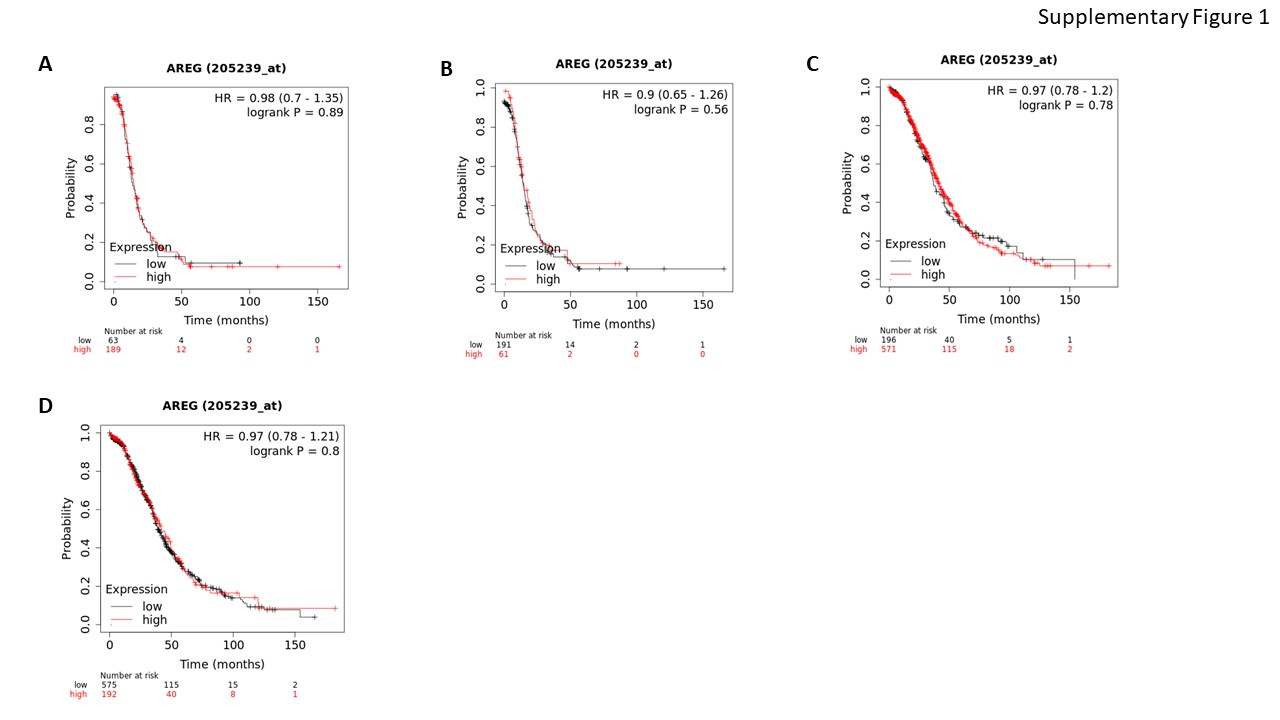

Supplement: Supplementary file 4 [file Image1.JPEG]

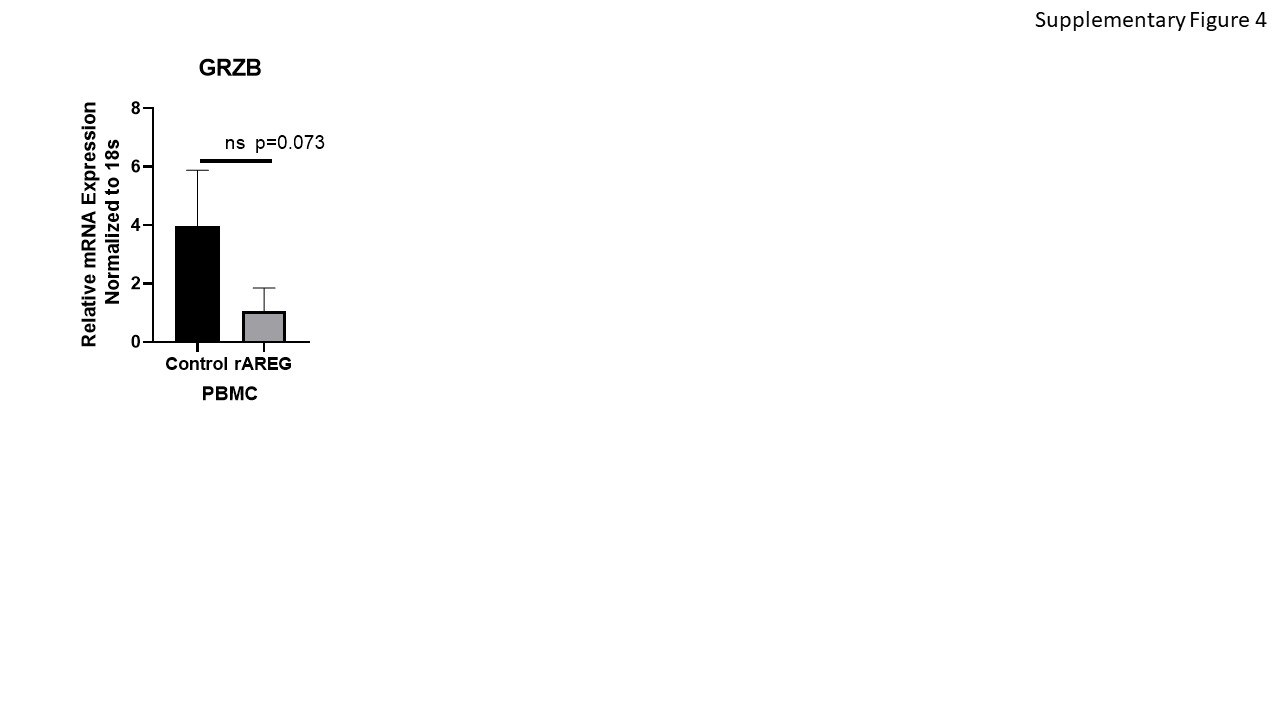

Supplement: Supplementary file 5 [file Image4.JPEG]

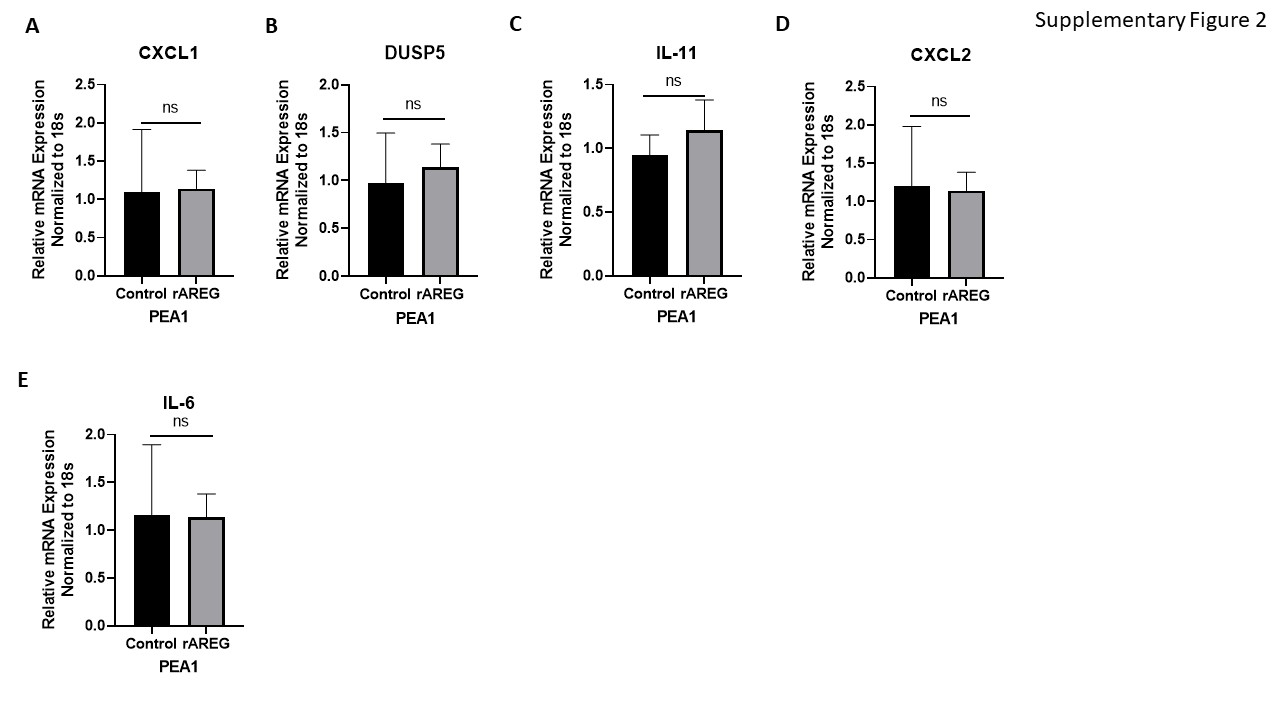

Supplement: Supplementary file 6 [file Image2.JPEG]
