# Supplementary material for: Enhanced amphiregulin exposure promotes modulation of the high grade serous ovarian cancer tumor immune microenvironment
Source: Front Pharmacol. 2024 May 20;15:1375421. doi: 10.3389/fphar.2024.1375421 (PMC11144882; doi:10.3389/fphar.2024.1375421)
Supplement: Supplementary file 3 [file Presentation1.PPTX]

## Slide 1
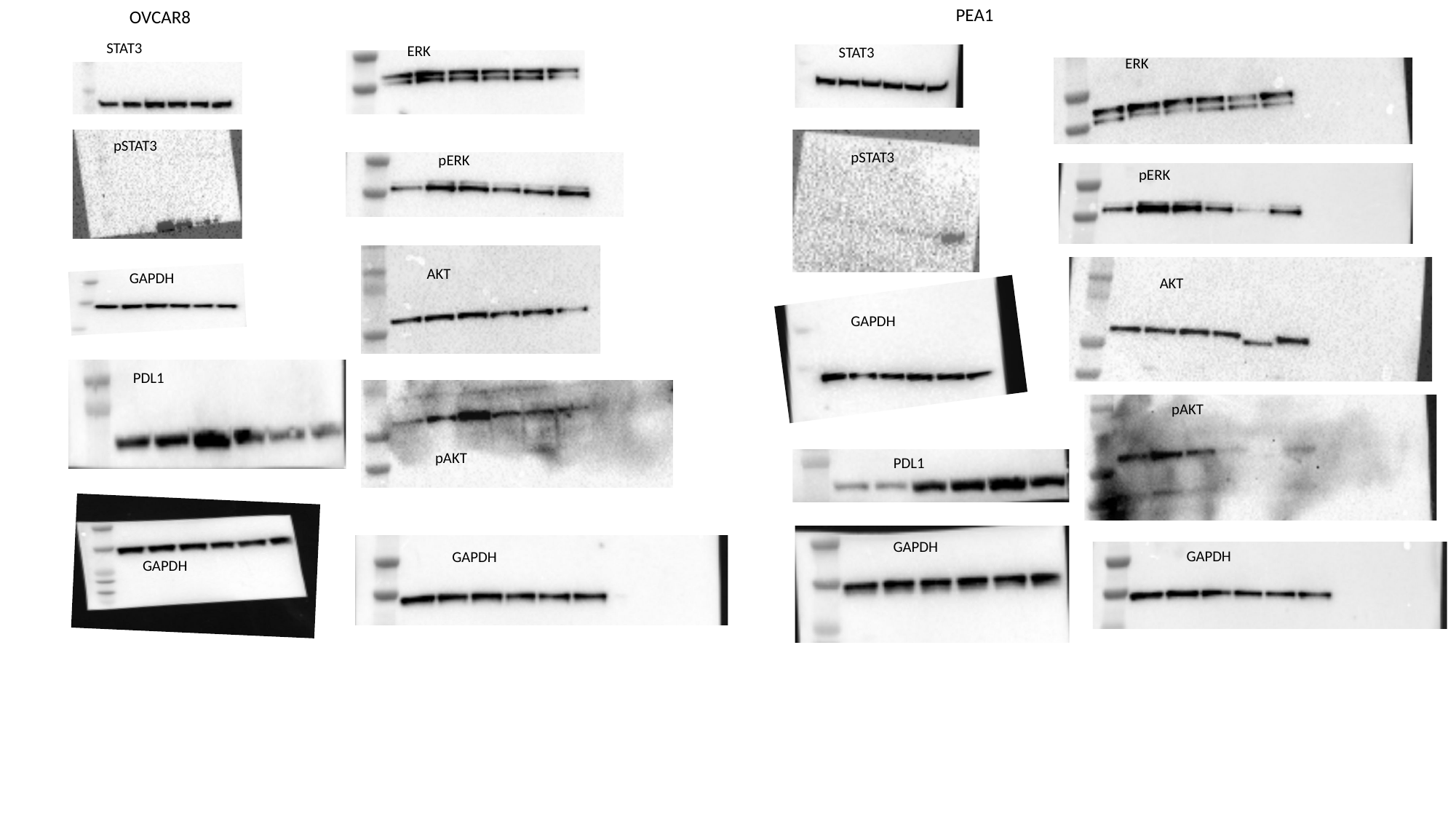

OVCAR8
PEA1
STAT3
ERK
STAT3
pSTAT3
GAPDH
PDL1
GAPDH
ERK
pSTAT3
pERK
pERK
AKT
GAPDH
AKT
PDL1
pAKT
pAKT
GAPDH
GAPDH
GAPDH
