# Supplementary material for: Enhanced amphiregulin exposure promotes modulation of the high grade serous ovarian cancer tumor immune microenvironment
Source: Front Pharmacol. 2024 May 20;15:1375421. doi: 10.3389/fphar.2024.1375421 (PMC11144882; doi:10.3389/fphar.2024.1375421)
Supplement: Supplementary file 7 [file Table2.DOCX]

Supplemental Table 2

Serum Multiplex Cytokine/Chemokine Analysis

|  | Saline Control Mean [range](pg/ml) | rAREG Mean[range] (pg/ml) | p-value |
| --- | --- | --- | --- |
| Eotaxin | 934.86 [784.59-1117.85] | 804.67 [301.24-1071.97] | 0.422 |
| G-CSF | 720.58 [303.48-1320.85] | 2285.16 [667.08-5178.77] | 0.104 |
| GM-CSF | 11.51 [0-19.85] | 0 [0-0] | 0.168 |
| IFNy | 3.63 [0-17.69] | 38.22 [0-191.11] | 0.394 |
| IL-1α | 639.12 [272.17-1302.92] | 548.38 [55.55-1084.92] | 0.725 |
| IL-1β | 14.13 [0.13-65.12] | 1.91 [0-3.01] | 0.367 |
| IL-2 | 8.27 [2.88-11.47] | 3.71 [0-5.7] | 0.097 |
| IL-3 | 0.48 [0.28-0.88] | 0.33 [0.18-0.63] | 0.291 |
| IL-4 | 0.194 [0-0.47] | 0.35 [0.2-0.52] | 0.152 |
| IL-5 | 16.55 [12.11-19.98] | 9.42 [5.15-13.30] | 0.001 |
| IL-6 | 44.52 [4.37-83.57] | 199.76 [7.64-517.41] | 0.145 |
| IL-7 | N/A under detection limit | N/A under detection limit |  |
| IL-9 | 1431.51 [0-6870.33] | 78.47 [33.62-108.21] | 0.409 |
| IL-10 | 11.57 [0-25.84] | 28.49 [0-79.19] | 0.330 |
| IL-12p40 | 7.31 [0-30.27] | 6.14 [0.84-14.64] | 0.858 |
| IL-12p70 | N/A under detection limit | N/A under detection limit |  |
| IL-13 | 75.22 [53.08-107.52] | 104.31 [78.83-117.0] | 0.053 |
| IL-15 | 4.78 [0-22.54] | 0.74 [0-3.71] | 0.396 |
| IL-17F | 1.05 [0.30-1.86]* | 1.13 [0.3-1.86]* | 0.744 |
| IP-10 | 173.77 [88.76-237.34] | 157.37 [128.74-195.77] | 0.619 |
| KC | 288.61 [146.01-525.68] | 193.45 [50.73-524.47 | 0.394 |
| LIF | 0 [0-0] | 0.44 [0-1.29] | 0.15 |
| LIX | 7780.24 [7447.62-8450.08] | 6977.07 [4495.24-8152.97] | 0.267 |
| M-CSF | N/A under detection limit | N/A under detection limit |  |
| MCP-1 | 50.20 [0.23-152.51] | 49.04 [0-111.66] | 0.972 |
| MIG | 1025.55 [594.82-1357.02] | 626.62 [113.45-1147.85] | 0.113 |
| MIP-1α | 104.01 [79.45-187.79] | 66.95 [53.19-79.45] | 0.124 |
| MIP-1β | 87.32 [12.6-144.59] | 41.93 [0-94.42] | 0.124 |
| MIP-2 | 288.474 [134.83-381.69] | 233.17 [0-349.62] | 0.51 |
| RANTES | 112.09 [65.8-199.84] | 126.16 [56.92-184.73] | 0.675 |
| TNFα | 5.53 [0-13.97] | 4.62 [0-10.44] | 0.787 |
| VEGF | 37.51 [0.91-132.83] | 219.48 [1.33-613.55] | 0.172 |
| 6Ckine/Exodus 2 | 850.88 [275.17-1288.30] | 716.74 [512.94-799.02] | 0.495 |
| EPO | 413.98 [168.43-1242.76] | 1146.75 [881.20-2543.78] | 0.129 |
| Fractalkine | 104.54 [65.98-141.28] | 105.80 [75.89-138.03] | 0.947 |
| IFNβ-1 | 103.00 [39.69-158.4] | 112.80 [92.73-161.46] | 0.688 |
| IL-11 | 20.69 [13.75-27.22] | 25.94 [12.6-42.78] | 0.37 |
| IL-16 | 3659.68 [1047.48-7352.97] | 3621.41 [2165.70-5186.64] | 0.978 |
| IL-20 | 70.11 [49.70-90.33] | 146.52 [11107-256.35] | 0.028 |
| MCP-5 | 172.36 [62.09-268.46] | 266.37 [82.85-646.35] | 0.41 |
| MDC | 193.63 [91.24-257.75] | 234.31 [140.89-313.15] | 0.376 |
| MIP-3α | 90.98 [41.78-158.10] | 97.25 [53.86-143.06] | 0.811 |
| MIP-3β | 62.49 [55.97-69.02] | 82.71 [57.94-122.87] | 0.107 |
| TARC | 180.89 [79.6-227.95] | 193.59 [131.18-278.97] | 0.749 |
| TIMP-1 | 54085.5 [2168.74-237827.34] | 22849.1 [6739.82-10039.6] | 0.535 |

*Extrapolated data. The signal was below the range of standard curve allowed for the estimation of the concentration value. Extrapolated values fall outside of the regression model and therefore may be less accurate.
